# Supplementary material for: Expanding Mouse-Adapted Yamagata-like Influenza B Viruses in Eggs Enhances In Vivo Lethality in BALB/c Mice
Source: Viruses. 2022 Jun 14;14(6):1299. doi: 10.3390/v14061299 (PMC9229684; doi:10.3390/v14061299)
Supplement: Supplementary file 1 [file viruses-14-01299-s001.zip › viruses-1761878-supplementary-update.pdf]

Figure S1. Hemagglutinin amino acid sequence alignment of B/Florida/06.

```
B_Florida_4_06_parent_HA      1  MKAIIVLLMVVTSNADRICTGITSSNSPHVVKTATQGEVNVTGVIPLTTTPTKSYFANLKGTRTRGKLCPDCLNCTDLDV  80
B_Florida_4_06_p10_cell_HA    1  MKAIIVLLMVVTSNADRICTGITSSNSPHVVKTATQGEVNVTGVIPLTTTPTKSYFANLKGTRTRGKLCPDCLNCTDLDV  80
B_Florida_4_06_p10_egg_HA     1  MKAIIVLLMVVTSNADRICTGITSSNSPHVVKTATQGEVNVTGVIPLTTTPTKSYFANLKGTRTRGKLCPDCLNCTDLDV  80
B_Florida_4_06_p20_cell_HA    1  MKAIIVLLMVVTSNADRICTGITSSNSPHVVKTATQGEVNVTGVIPLTTTPTKSYFANLKGTRTRGKLCPDCLNCTDLDV  80
B_Florida_4_06_p20_egg_HA     1  MKAIIVLLMVVTSNADRICTGITSSNSPHVVKTATQGEVNVTGVIPLTTTPTKSYFANLKGTRTRGKLCPDCLNCTDLDV  80
                                 MKAIIVLLMVVTSNADRICTGITSSNSPHVVKTATQGEVNVTGVIPLTTTPTKSYFANLKGTRTRGKLCPDCLNCTDLDV

B_Florida_4_06_parent_HA     81  ALGRPMCVGTTPSAKASILHEVKPVTSGCFPIMHDRTKIRQLPNLLRGYENIRLSTQNVIDAEKAPGGPYRLGTSGSCPN  160
B_Florida_4_06_p10_cell_HA   81  ALGRPMCVGTTPSAKASILHEVKPVTSGCFPIMHDRTKIRQLPNLLRGYENIRLSTQNVIDAEKAPGGPYRLGTSGSCPN  160
B_Florida_4_06_p10_egg_HA    81  ALGRPMCVGTTPSAKASILHEVKPVTSGCFPIMHDRTKIRQLPNLLRGYENIRLSTQNVIDAEKAPGGPYRLGTSGSCPN  160
B_Florida_4_06_p20_cell_HA   81  ALGRPMCVGTTPSAKASILHEVKPVTSGCFPIMHDRTKIRQLPNLLRGYENIRLSTQNVIDAEKAPGGPYRLGTSGSCPN  160
B_Florida_4_06_p20_egg_HA    81  ALGRPMCVGTTPSAKASILHEVKPVTSGCFPIMHDRTKIRQLPNLLRGYENIRLSTQNVIDAEKAPGGPYRLGTSGSCPN  160
                                 ALGRPMCVGTTPSAKASILHEVKPVTSGCFPIMHDRTKIRQLPNLLRGYENIRLSTQNVIDAEKAPGGPYRLGTSGSCPN

B_Florida_4_06_parent_HA    161  ATSKSGFFATMAWAVPKDNNKNATNPLTVEVPYICTEGEDQITVWGFHSDDKTQMKNLYGDSNPQKFTSSANGVTTHYVS  240
B_Florida_4_06_p10_cell_HA  161  ATSKSGFFATMAWAVPKDNNKNATNPLTVEVPYICTEGEDQITVWGFHSDDKTQMKNLYGDSNPQKFTSSANGVTTHYVS  240
B_Florida_4_06_p10_egg_HA   161  ATSKSGFFATMAWAVPKDNNKNATNPLTVEVPYICTEGEDQITVWGFHSDDKTQMKNLYGDSNPQKFTSSANGVTTHYVS  240
B_Florida_4_06_p20_cell_HA  161  ATSKSGFFATMAWAVPKDNNKNATNPLTVEVPYICTEGEDQITVWGFHSDDKTQMKNLYGDSNPQKFTSSANGVTTHYVS  240
B_Florida_4_06_p20_egg_HA   161  ATSKSGFFATMAWAVPKDNNKNATNPLTVEVPYICTEGEDQITVWGFHSDDKTQMKNLYGDSNPQKFTSSANGVTTHYVS  240
                                 ATSKSGFFATMAWAVPKDNNKNATNPLTVEVPYICTEGEDQITVWGFHSDDKTQMKNLYGDSNPQKFTSSANGVTTHYVS

B_Florida_4_06_parent_HA    241  QIGSFPDQTEDGGLPQSGRIVVDYMMQKPGKTGTIVYQRGVLLPQKVWCASGRSKVIKGSLPLIGEADCLHEKYGGLNKS  320
B_Florida_4_06_p10_cell_HA  241  QIGSFPDQTEDGGLPQSGRIVVDYMMQKPGKTGTIVYQRGVLLPQKVWCASGRSKVIKGSLPLIGEADCLHEKYGGLNKS  320
B_Florida_4_06_p10_egg_HA   241  QIGSFPDQTEDGGLPQSGRIVVDYMMQKPGKTGTIVYQRGVLLPQKVWCASGRSKVIKGSLPLIGEADCLHEKYGGLNKS  320
B_Florida_4_06_p20_cell_HA  241  QIGSFPDQTEDGGLPQSGRIVVDYMMQKPGKTGTIVYQRGVLLPQKVWCASGRSKVIKGSLPLIGEADCLHEKYGGLNKS  320
B_Florida_4_06_p20_egg_HA   241  QIGSFPDQTEDGGLPQSGRIVVDYMMQKPGKTGTIVYQRGVLLPQKVWCASGRSKVIKGSLPLIGEADCLHEKYGGLNKS  320
                                 QIGSFPDQTEDGGLPQSGRIVVDYMMQKPGKTGTIVYQRGVLLPQKVWCASGRSKVIKGSLPLIGEADCLHEKYGGLNKS

B_Florida_4_06_parent_HA    321  KPYYTGEHAKAIGNCPIWVKTPLKLANGTKYRPPAKLLKERGFFGAIAGFLEGGWEGMIAGWHGYTSHGAHGVAVAADLK  400
B_Florida_4_06_p10_cell_HA  321  KPYYTGEHAKAIGNCPIWVKTPLKLANGTKYRPPAKLLKERGFFGAIAGFLEGGWEGMIAGWHGYTSHGAHGVAVAADLK  400
B_Florida_4_06_p10_egg_HA   321  KPYYTGEHAKAIGNCPIWVKTPLKLANGTKYRPPAKLLKERGFFGAIAGFLEGGWEGMIAGWHGYTSHGAHGVAVAADLK  400
B_Florida_4_06_p20_cell_HA  321  KPYYTGEHAKAIGNCPIWVKTPLKLANGTKYRPPAKLLKERGFFGAIAGFLEGGWEGMIAGWHGYTSHGAHGVAVAADLK  400
B_Florida_4_06_p20_egg_HA   321  KPYYTGEHAKAIGNCPIWVKTPLKLANGTKYRPPAKLLKERGFFGAIAGFLEGGWEGMIAGWHGYTSHGAHGVAVAADLK  400
                                 KPYYTGEHAKAIGNCPIWVKTPLKLANGTKYRPPAKLLKERGFFGAIAGFLEGGWEGMIAGWHGYTSHGAHGVAVAADLK

B_Florida_4_06_parent_HA    401  STQEAINKITKNLNSLSELEVKNLQRLSGAMDELHNEILELDEKVDDLRADTISSQIELAVLLSNEGIINSEDEHLLALE  480
B_Florida_4_06_p10_cell_HA  401  STQEAINKITKNLNSLSELEVKNLQRLSGAMDELHNEILELDEKVDDLRADTISSQIELAVLLSNEGIINSEDEHLLALE  480
B_Florida_4_06_p10_egg_HA   401  STQEAINKITKNLNSLSELEVKNLQRLSGAMDELHNEILELDEKVDDLRADTISSQIELAVLLSNEGIINSEDEHLLALE  480
B_Florida_4_06_p20_cell_HA  401  STQEAINKITKNLNSLSELEVKNLQRLSGAMDELHNEILELDEKVDDLRADTISSQIELAVLLSNEGIINSEDEHLLALE  480
B_Florida_4_06_p20_egg_HA   401  STQEAINKITKNLNSLSELEVKNLQRLSGAMDELHNEILELDEKVDDLRADTISSQIELAVLLSNEGIINSEDEHLLALE  480
                                 STQEAINKITKNLNSLSELEVKNLQRLSGAMDELHNEILELDEKVDDLRADTISSQIELAVLLSNEGIINSEDEHLLALE

B_Florida_4_06_parent_HA    481  RKLKKMLGPSAVEIGNGCFETKHKCNQTCLDRIAAGTFNAGEFSLPTFDSLNITAASLNDDGLDNHTILLYYSTAASSLA  560
B_Florida_4_06_p10_cell_HA  481  RKLKKMLGPSAVEIGNGCFETKHKCNQTCLDRIAAGTFNAGEFSLPTFDSLNITAASLNDDGLDNHTILLYYSTAASSLA  560
B_Florida_4_06_p10_egg_HA   481  RKLKKMLGPSAVEIGNGCFETKHKCNQTCLDRIAAGTFNAGEFSLPTFDSLNITAASLNDDGLDNHTILLYYSTAASSLA  560
B_Florida_4_06_p20_cell_HA  481  RKLKKMLGPSAVEIGNGCFETKHKCNQTCLDRIAAGTFNAGEFSLPTFDSLNITAASLNDDGLDNHTILLYYSTAASSLA  560
B_Florida_4_06_p20_egg_HA   481  RKLKKMLGPSAVEIGNGCFETKHKCNQTCLDRIAAGTFNAGEFSLPTFDSLNITAASLNDDGLDNHTILLYYSTAASSLA  560
                                 RKLKKMLGPSAVEIGNGCFETKHKCNQTCLDRIAAGTFNAGEFSLPTFDSLNITAASLNDDGLDNHTILLYYSTAASSLA

B_Florida_4_06_parent_HA    561  VTLMLAIFIVYMVSRDNVSCSICL*  585
B_Florida_4_06_p10_cell_HA  561  VTLMLAIFIVYMVSRDNVSCSICL*  585
B_Florida_4_06_p10_egg_HA   561  VTLMLAIFIVYMVSRDNVSCSICL*  585
B_Florida_4_06_p20_cell_HA  561  VTLMLAIFIVYMVSRDNVSCSICL*  585
B_Florida_4_06_p20_egg_HA   561  VTLMLAIFIVYMVSRDNVSCSICL*  585
                                 VTLMLAIFIVYMVSRDNVSCSICL
```

Figure S2. Hemagglutinin nucleotide sequence alignment of B/Florida/06.

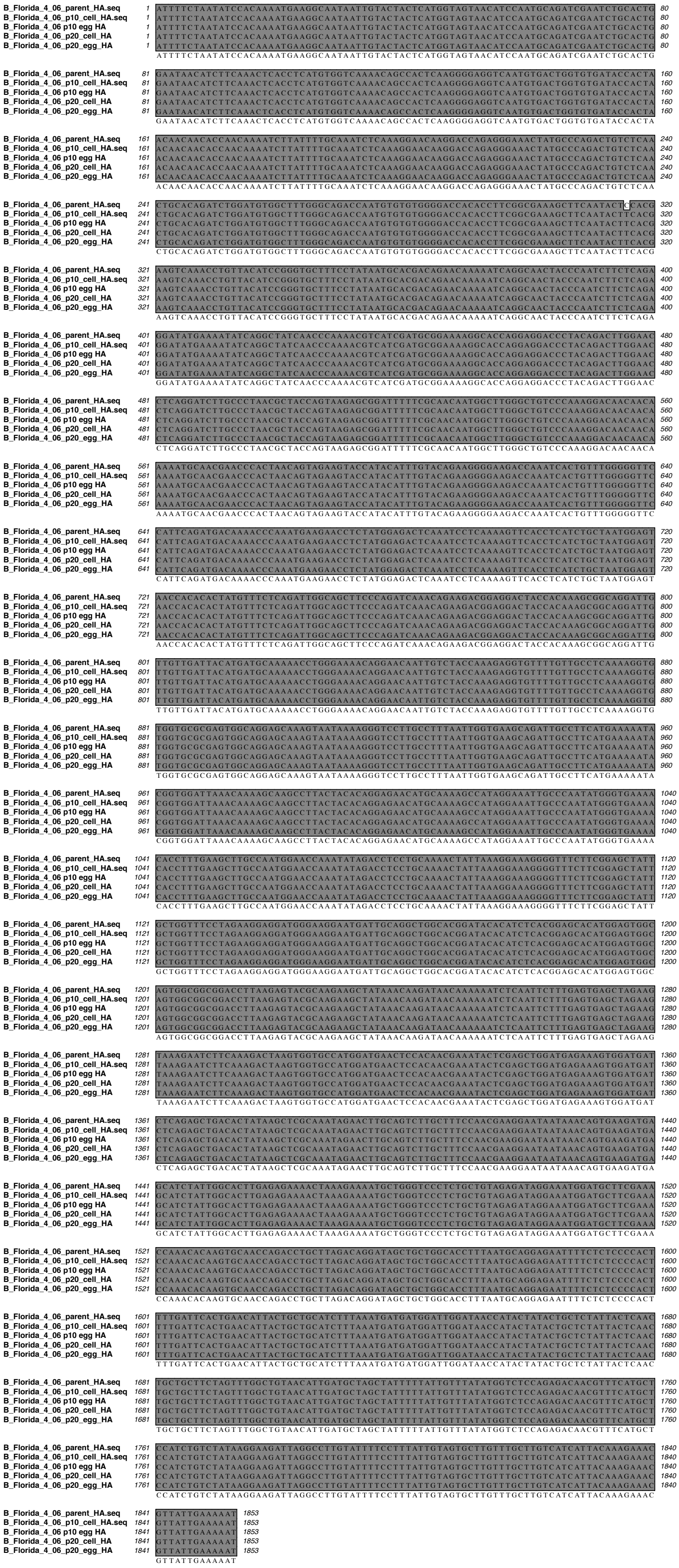

Figure S3. Neuraminidase amino acid sequence alignment of B/Florida/06.

```
B_Florida_4_06_parent_NA    1  MLPSTIQTLTLFLTSGGVLLSLYVSASLSYLLYSDILLKFSQTEITAPIMPLDCANASNVQAVNRSAAKGVTLLLPEPEW  80
B_Florida_4_06 p10 cell NA  1  MLPSTIQTLTLFLTSGGVLLSLYVSASLSYLLYSDILLKFSQTEITAPIMPLDCANASNVQAVNRSAAKGVTLLLPEPEW  80
B_Florida_4_06_p10_egg_NA   1  MLPSTIQTLTLFLTSGGVLLSLYVSASLSYLLYSDILLKFSQTEITAPIMPLDCANASNVQAVNRSAAKGVTLLLPEPEW  80
B_Florida_4_06_p20_cell_NA  1  MLPSTIQTLTLFLTSGGVLLSLYVSASLSYLLYSDILLKFSQTEITAPIMPLDCANASNVQAVNRSAAKGVTLLLPEPEW  80
B_Florida_4_06_p20_egg_NA   1  MLPSTIQTLTLFLTSGGVLLSLYVSASLSYLLYSDILLKFSQTEITAPIMPLDCANASNVQAVNRSAAKGVTLLLPEPEW  80
                               MLPSTIQTLTLFLTSGGVLLSLYVSASLSYLLYSDILLKFSQTEITAPIMPLDCANASNVQAVNRSAAKGVTLLLPEPEW

B_Florida_4_06_parent_NA    81  TYPRLSCPGSTFQKALLISPHRFGETKGNSAPLIIREPFIACGPTECKHFALTHYAAQPGGYYNGTREDRNKLRHLISVK  160
B_Florida_4_06 p10 cell NA  81  TYPRLSCPGSTFQKALLISPHRFGETKGNSAPLIIREPFIACGPTECKHFALTHYAAQPGGYYNGTREDRNKLRHLISVK  160
B_Florida_4_06_p10_egg_NA   81  TYPRLSCPGSTFQKALLISPHRFGETKGNSAPLIIREPFIACGPTECKHFALTHYAAQPGGYYNGTREDRNKLRHLISVK  160
B_Florida_4_06_p20_cell_NA  81  TYPRLSCPGSTFQKALLISPHRFGETKGNSAPLIIREPFIACGPTECKHFALTHYAAQPGGYYNGTREDRNKLRHLISVK  160
B_Florida_4_06_p20_egg_NA   81  TYPRLSCPGSTFQKALLISPHRFGETKGNSAPLIIREPFIACGPTECKHFALTHYAAQPGGYYNGTREDRNKLRHLISVK  160
                                TYPRLSCPGSTFQKALLISPHRFGETKGNSAPLIIREPFIACGPTECKHFALTHYAAQPGGYYNGTREDRNKLRHLISVK

B_Florida_4_06_parent_NA    161  LGKIPTVENSIFHMAAWSGSACHDGKEWTYIGVDGPDSNALLKIKYGEAYTDTYHSYAKNILRTQESACNCIGGDCYLMI  240
B_Florida_4_06 p10 cell NA  161  LGKIPTVENSIFHMAAWSGSACHDGKEWTYIGVDGPDSNALLKIKYGEAYTDTYHSYAKNILRTQESACNCIGGDCYLMI  240
B_Florida_4_06_p10_egg_NA   161  LGKIPTVENSIFHMAAWSGSACHDGKEWTYIGVDGPDSNALLKIKYGEAYTDTYHSYAKNILRTQESACNCIGGDCYLMI  240
B_Florida_4_06_p20_cell_NA  161  LGKIPTVENSIFHMAAWSGSACHDGKEWTYIGVDGPDSNALLKIKYGEAYTDTYHSYAKNILRTQESACNCIGGDCYLMI  240
B_Florida_4_06_p20_egg_NA   161  LGKIPTVENSIFHMAAWSGSACHDGKEWTYIGVDGPDSNALLKIKYGEAYTDTYHSYAKNILRTQESACNCIGGDCYLMI  240
                                 LGKIPTVENSIFHMAAWSGSACHDGKEWTYIGVDGPDSNALLKIKYGEAYTDTYHSYAKNILRTQESACNCIGGDCYLMI

B_Florida_4_06_parent_NA    241  TDGPASGISECRFLKIREGRIIKEIFPTGRVKHTEECTCGFASNKTIECACRDNSYTAKRPFVKLNVETDTAEIRLMCTE  320
B_Florida_4_06 p10 cell NA  241  TDGPASGISECRFLKIREGRIIKEIFPTGRVKHTEECTCGFASNKTIECACRDNSYTAKRPFVKLNVETDTAEIRLMCTE  320
B_Florida_4_06_p10_egg_NA   241  TDGPASGISECRFLKIREGRIIKEIFPTGRVKHTEECTCGFASNKTIECACRDNSYTAKRPFVKLNVETDTAEIRLMCTE  320
B_Florida_4_06_p20_cell_NA  241  TDGPASGISECRFLKIREGRIIKEIFPTGRVKHTEECTCGFASNKTIECACRDNSYTAKRPFVKLNVETDTAEIRLMCTE  320
B_Florida_4_06_p20_egg_NA   241  TDGPASGISECRFLKIREGRIIKEIFPTGRVKHTEECTCGFASNKTIECACRDNSYTAKRPFVKLNVETDTAEIRLMCTE  320
                                 TDGPASGISECRFLKIREGRIIKEIFPTGRVKHTEECTCGFASNKTIECACRDNSYTAKRPFVKLNVETDTAEIRLMCTE

B_Florida_4_06_parent_NA    321  TYLDTPRPNDGSITGPCESDGDKGSGGIKGGFVHQRMASKIGRWYSRTMSKTKRMGMGLYVKYDGDPWTDSEALALSGVM  400
B_Florida_4_06 p10 cell NA  321  TYLDTPRPNDGSITGPCESDGDKGSGGIKGGFVHQRMASKIGRWYSRTMSKTKRMGMGLYVKYDGDPWTDSEALALSGVM  400
B_Florida_4_06_p10_egg_NA   321  TYLDTPRPNDGSITGPCESDGDKGSGGIKGGFVHQRMASKIGRWYSRTMSKTKRMGMGLYVKYDGDPWTDSEALALSGVM  400
B_Florida_4_06_p20_cell_NA  321  TYLDTPRPNDGSITGPCESDGDKGSGGIKGGFVHQRMASKIGRWYSRTMSKTKRMGMGLYVKYDGDPWTDSEALALSGVM  400
B_Florida_4_06_p20_egg_NA   321  TYLDTPRPNDGSITGPCESDGDKGSGGIKGGFVHQRMASKIGRWYSRTMSKTKRMGMGLYVKYDGDPWTDSEALALSGVM  400
                                 TYLDTPRPNDGSITGPCESDGDKGSGGIKGGFVHQRMASKIGRWYSRTMSKTKRMGMGLYVKYDGDPWTDSEALALSGVM

B_Florida_4_06_parent_NA    401  VSMEEPGWYSFGFEIKDKKCDVPCIGIEMVHDGGKTTWHSAATAIYCLMGSGQLLWDTVTGVDMAL*  467
B_Florida_4_06 p10 cell NA  401  VSMEEPGWYSFGFEIKDKKCDVPCIGIEMVHDGGKTTWHSAATAIYCLMGSGQLLWDTVTGVDMAL*  467
B_Florida_4_06_p10_egg_NA   401  VSMEEPGWYSFGFEIKDKKCDVPCIGIEMVHDGGKTTWHSAATAIYCLMGSGQLLWDTVTGVDMAL*  467
B_Florida_4_06_p20_cell_NA  401  VSMEEPGWYSFGFEIKDKKCDVPCIGIEMVHDGGKTTWHSAATAIYCLMGSGQLLWDTVTGVDMAL*  467
B_Florida_4_06_p20_egg_NA   401  VSMEEPGWYSFGFEIKDKKCDVPCIGIEMVHDGGKTTWHSAATAIYCLMGSGQLLWDTVTGVDMAL*  467
                                 VSMEEPGWYSFGFEIKDKKCDVPCIGIEMVHDGGKTTWHSAATAIYCLMGSGQLLWDTVTGVDMAL
```

Figure S4. Neuraminidase nucleotide sequence alignment of B/Florida/06.

```
B_Florida_4_06_parent_NA       1 ATCTTCTCAAAACTGAGGCAAATAGGCCAAAAATGAACAATGCTACCTTCAACTATACAAACGTTAACCCTATTTCTCAC 80
B_Florida_4_06 p10 cell NA     1 ATCTTCTCAAAACTGAGGCAAATAGGCCAAAAATGAACAATGCTACCTTCAACTATACAAACGTTAACCCTATTTCTCAC 80
B_Florida_4_06_p10_egg_NA.seq  1 ATCTTCTCAAAACTGAGGCAAATAGGCCAAAAATGAACAATGCTACCTTCAACTATACAAACGTTAACCCTATTTCTCAC 80
B_Florida_4_06 p20 cell NA     1 ATCTTCTCAAAACTGAGGCAAATAGGCCAAAAATGAACAATGCTACCTTCAACTATACAAACGTTAACCCTATTTCTCAC 80
B_Florida_4_06_p20_egg_NA      1 ATCTTCTCAAAACTGAGGCAAATAGGCCAAAAATGAACAATGCTACCTTCAACTATACAAACGTTAACCCTATTTCTCAC 80
                                 ATCTTCTCAAAACTGAGGCAAATAGGCCAAAAATGAACAATGCTACCTTCAACTATACAAACGTTAACCCTATTTCTCAC

B_Florida_4_06_parent_NA      81 ATCAGGGGGAGTGTTATTATCACTATATGTGTCAGCTTCATTGTCATACTTACTATATTCGGATATATTGCTAAAATTTT 160
B_Florida_4_06 p10 cell NA    81 ATCAGGGGGAGTGTTATTATCACTATATGTGTCAGCTTCATTGTCATACTTACTATATTCGGATATATTGCTAAAATTTT 160
B_Florida_4_06_p10_egg_NA.seq 81 ATCAGGGGGAGTGTTATTATCACTATATGTGTCAGCTTCATTGTCATACTTACTATATTCGGATATATTGCTAAAATTTT 160
B_Florida_4_06 p20 cell NA    81 ATCAGGGGGAGTGTTATTATCACTATATGTGTCAGCTTCATTGTCATACTTACTATATTCGGATATATTGCTAAAATTTT 160
B_Florida_4_06_p20_egg_NA     81 ATCAGGGGGAGTGTTATTATCACTATATGTGTCAGCTTCATTGTCATACTTACTATATTCGGATATATTGCTAAAATTTT 160
                                 ATCAGGGGGAGTGTTATTATCACTATATGTGTCAGCTTCATTGTCATACTTACTATATTCGGATATATTGCTAAAATTTT

B_Florida_4_06_parent_NA      161 CACAAACAGAAATAACTGCACCAATAATGCCATTGGATTGTGCAAACGCATCAAATGTTCAGGCTGTGAACCGTTCTGCA 240
B_Florida_4_06 p10 cell NA    161 CACAAACAGAAATAACTGCACCAATAATGCCATTGGATTGTGCAAACGCATCAAATGTTCAGGCTGTGAACCGTTCTGCA 240
B_Florida_4_06_p10_egg_NA.seq 161 CACAAACAGAAATAACTGCACCAATAATGCCATTGGATTGTGCAAACGCATCAAATGTTCAGGCTGTGAACCGTTCTGCA 240
B_Florida_4_06 p20 cell NA    161 CACAAACAGAAATAACTGCACCAATAATGCCATTGGATTGTGCAAACGCATCAAATGTTCAGGCTGTGAACCGTTCTGCA 240
B_Florida_4_06_p20_egg_NA     161 CACAAACAGAAATAACTGCACCAATAATGCCATTGGATTGTGCAAACGCATCAAATGTTCAGGCTGTGAACCGTTCTGCA 240
                                  CACAAACAGAAATAACTGCACCAATAATGCCATTGGATTGTGCAAACGCATCAAATGTTCAGGCTGTGAACCGTTCTGCA

B_Florida_4_06_parent_NA      241 GCAAAAGGGGTGACACTTCTTCTCCCAGAACCGGAGTGGACATACCCTCGTTTATCTTGCCCGGGCTCAACCTTTCAGAA 320
B_Florida_4_06 p10 cell NA    241 GCAAAAGGGGTGACACTTCTTCTCCCAGAACCGGAGTGGACATACCCTCGTTTATCTTGCCCGGGCTCAACCTTTCAGAA 320
B_Florida_4_06_p10_egg_NA.seq 241 GCAAAAGGGGTGACACTTCTTCTCCCAGAACCGGAGTGGACATACCCTCGTTTATCTTGCCCGGGCTCAACCTTTCAGAA 320
B_Florida_4_06 p20 cell NA    241 GCAAAAGGGGTGACACTTCTTCTCCCAGAACCGGAGTGGACATACCCTCGTTTATCTTGCCCGGGCTCAACCTTTCAGAA 320
B_Florida_4_06_p20_egg_NA     241 GCAAAAGGGGTGACACTTCTTCTCCCAGAACCGGAGTGGACATACCCTCGTTTATCTTGCCCGGGCTCAACCTTTCAGAA 320
                                  GCAAAAGGGGTGACACTTCTTCTCCCAGAACCGGAGTGGACATACCCTCGTTTATCTTGCCCGGGCTCAACCTTTCAGAA

B_Florida_4_06_parent_NA      321 AGCACTCCTAATTAGCCCCCATAGATTCGGAGAAACCAAAGGAAACTCAGCTCCCTTGATAATAAGGGAACCTTTTATTG 400
B_Florida_4_06 p10 cell NA    321 AGCACTCCTAATTAGCCCCCATAGATTCGGAGAAACCAAAGGAAACTCAGCTCCCTTGATAATAAGGGAACCTTTTATTG 400
B_Florida_4_06_p10_egg_NA.seq 321 AGCACTCCTAATTAGCCCCCATAGATTCGGAGAAACCAAAGGAAACTCAGCTCCCTTGATAATAAGGGAACCTTTTATTG 400
B_Florida_4_06 p20 cell NA    321 AGCACTCCTAATTAGCCCCCATAGATTCGGAGAAACCAAAGGAAACTCAGCTCCCTTGATAATAAGGGAACCTTTTATTG 400
B_Florida_4_06_p20_egg_NA     321 AGCACTCCTAATTAGCCCCCATAGATTCGGAGAAACCAAAGGAAACTCAGCTCCCTTGATAATAAGGGAACCTTTTATTG 400
                                  AGCACTCCTAATTAGCCCCCATAGATTCGGAGAAACCAAAGGAAACTCAGCTCCCTTGATAATAAGGGAACCTTTTATTG

B_Florida_4_06_parent_NA      401 CTTGTGGACCAACGGAATGCAAACACTTTGCTCTAACCCATTATGCAGCTCAACCAGGGGGATACTACAATGGAACAAGA 480
B_Florida_4_06 p10 cell NA    401 CTTGTGGACCAACGGAATGCAAACACTTTGCTCTAACCCATTATGCAGCTCAACCAGGGGGATACTACAATGGAACAAGA 480
B_Florida_4_06_p10_egg_NA.seq 401 CTTGTGGACCAACGGAATGCAAACACTTTGCTCTAACCCATTATGCAGCTCAACCAGGGGGATACTACAATGGAACAAGA 480
B_Florida_4_06 p20 cell NA    401 CTTGTGGACCAACGGAATGCAAACACTTTGCTCTAACCCATTATGCAGCTCAACCAGGGGGATACTACAATGGAACAAGA 480
B_Florida_4_06_p20_egg_NA     401 CTTGTGGACCAACGGAATGCAAACACTTTGCTCTAACCCATTATGCAGCTCAACCAGGGGGATACTACAATGGAACAAGA 480
                                  CTTGTGGACCAACGGAATGCAAACACTTTGCTCTAACCCATTATGCAGCTCAACCAGGGGGATACTACAATGGAACAAGA

B_Florida_4_06_parent_NA      481 GAAGACAGAAACAAGCTGAGGCATCTAATTTCAGTCAAATTGGGCAAAATCCCAACAGTAGAAAACTCCATTTTCCATAT 560
B_Florida_4_06 p10 cell NA    481 GAAGACAGAAACAAGCTGAGGCATCTAATTTCAGTCAAATTGGGCAAAATCCCAACAGTAGAAAACTCCATTTTCCATAT 560
B_Florida_4_06_p10_egg_NA.seq 481 GAAGACAGAAACAAGCTGAGGCATCTAATTTCAGTCAAATTGGGCAAAATCCCAACAGTAGAAAACTCCATTTTCCATAT 560
B_Florida_4_06 p20 cell NA    481 GAAGACAGAAACAAGCTGAGGCATCTAATTTCAGTCAAATTGGGCAAAATCCCAACAGTAGAAAACTCCATTTTCCATAT 560
B_Florida_4_06_p20_egg_NA     481 GAAGACAGAAACAAGCTGAGGCATCTAATTTCAGTCAAATTGGGCAAAATCCCAACAGTAGAAAACTCCATTTTCCATAT 560
                                  GAAGACAGAAACAAGCTGAGGCATCTAATTTCAGTCAAATTGGGCAAAATCCCAACAGTAGAAAACTCCATTTTCCATAT

B_Florida_4_06_parent_NA      561 GGCAGCTTGGAGCGGGTCCGCATGCCATGATGGTAAAGAATGGACATATATCGGAGTTGATGGCCCCGACAGTAATGCAT 640
B_Florida_4_06 p10 cell NA    561 GGCAGCTTGGAGCGGGTCCGCATGCCATGATGGTAAAGAATGGACATATATCGGAGTTGATGGCCCCGACAGTAATGCAT 640
B_Florida_4_06_p10_egg_NA.seq 561 GGCAGCTTGGAGCGGGTCCGCATGCCATGATGGTAAAGAATGGACATATATCGGAGTTGATGGCCCCGACAGTAATGCAT 640
B_Florida_4_06 p20 cell NA    561 GGCAGCTTGGAGCGGGTCCGCATGCCATGATGGTAAAGAATGGACATATATCGGAGTTGATGGCCCCGACAGTAATGCAT 640
B_Florida_4_06_p20_egg_NA     561 GGCAGCTTGGAGCGGGTCCGCATGCCATGATGGTAAAGAATGGACATATATCGGAGTTGATGGCCCCGACAGTAATGCAT 640
                                  GGCAGCTTGGAGCGGGTCCGCATGCCATGATGGTAAAGAATGGACATATATCGGAGTTGATGGCCCCGACAGTAATGCAT

B_Florida_4_06_parent_NA      641 TACTCAAAATAAAATATGGAGAAGCATATACTGACACATACCATTCCTATGCAAAAAACATCCTAAGGACACAAGAAAGT 720
B_Florida_4_06 p10 cell NA    641 TACTCAAAATAAAATATGGAGAAGCATATACTGACACATACCATTCCTATGCAAAAAACATCCTAAGGACACAAGAAAGT 720
B_Florida_4_06_p10_egg_NA.seq 641 TACTCAAAATAAAATATGGAGAAGCATATACTGACACATACCATTCCTATGCAAAAAACATCCTAAGGACACAAGAAAGT 720
B_Florida_4_06 p20 cell NA    641 TACTCAAAATAAAATATGGAGAAGCATATACTGACACATACCATTCCTATGCAAAAAACATCCTAAGGACACAAGAAAGT 720
B_Florida_4_06_p20_egg_NA     641 TACTCAAAATAAAATATGGAGAAGCATATACTGACACATACCATTCCTATGCAAAAAACATCCTAAGGACACAAGAAAGT 720
                                  TACTCAAAATAAAATATGGAGAAGCATATACTGACACATACCATTCCTATGCAAAAAACATCCTAAGGACACAAGAAAGT

B_Florida_4_06_parent_NA      721 GCCTGCAATTGCATCGGGGGAGATTGTTATCTTATGATAACTGATGGCCCAGCTTCAGGGATTAGTGAATGCAGATTCCT 800
B_Florida_4_06 p10 cell NA    721 GCCTGCAATTGCATCGGGGGAGATTGTTATCTTATGATAACTGATGGCCCAGCTTCAGGGATTAGTGAATGCAGATTCCT 800
B_Florida_4_06_p10_egg_NA.seq 721 GCCTGCAATTGCATCGGGGGAGATTGTTATCTTATGATAACTGATGGCCCAGCTTCAGGGATTAGTGAATGCAGATTCCT 800
B_Florida_4_06 p20 cell NA    721 GCCTGCAATTGCATCGGGGGAGATTGTTATCTTATGATAACTGATGGCCCAGCTTCAGGGATTAGTGAATGCAGATTCCT 800
B_Florida_4_06_p20_egg_NA     721 GCCTGCAATTGCATCGGGGGAGATTGTTATCTTATGATAACTGATGGCCCAGCTTCAGGGATTAGTGAATGCAGATTCCT 800
                                  GCCTGCAATTGCATCGGGGGAGATTGTTATCTTATGATAACTGATGGCCCAGCTTCAGGGATTAGTGAATGCAGATTCCT

B_Florida_4_06_parent_NA      801 TAAGATTCGAGAGGGCCGAATAATAAAAGAAATATTTCCAACAGGAAGAGTAAAACATACTGAGGAATGCACATGCGGAT 880
B_Florida_4_06 p10 cell NA    801 TAAGATTCGAGAGGGCCGAATAATAAAAGAAATATTTCCAACAGGAAGAGTAAAACATACTGAGGAATGCACATGCGGAT 880
B_Florida_4_06_p10_egg_NA.seq 801 TAAGATTCGAGAGGGCCGAATAATAAAAGAAATATTTCCAACAGGAAGAGTAAAACATACTGAGGAATGCACATGCGGAT 880
B_Florida_4_06 p20 cell NA    801 TAAGATTCGAGAGGGCCGAATAATAAAAGAAATATTTCCAACAGGAAGAGTAAAACATACTGAGGAATGCACATGCGGAT 880
B_Florida_4_06_p20_egg_NA     801 TAAGATTCGAGAGGGCCGAATAATAAAAGAAATATTTCCAACAGGAAGAGTAAAACATACTGAGGAATGCACATGCGGAT 880
                                  TAAGATTCGAGAGGGCCGAATAATAAAAGAAATATTTCCAACAGGAAGAGTAAAACATACTGAGGAATGCACATGCGGAT

B_Florida_4_06_parent_NA      881 TTGCCAGCAACAAAACCATAGAATGTGCTTGTAGAGATAACAGTTACACAGCAAAAAGACCCTTTGTCAAATTAAATGTG 960
B_Florida_4_06 p10 cell NA    881 TTGCCAGCAACAAAACCATAGAATGTGCTTGTAGAGATAACAGTTACACAGCAAAAAGACCCTTTGTCAAATTAAATGTG 960
B_Florida_4_06_p10_egg_NA.seq 881 TTGCCAGCAACAAAACCATAGAATGTGCTTGTAGAGATAACAGTTACACAGCAAAAAGACCCTTTGTCAAATTAAATGTG 960
B_Florida_4_06 p20 cell NA    881 TTGCCAGCAACAAAACCATAGAATGTGCTTGTAGAGATAACAGTTACACAGCAAAAAGACCCTTTGTCAAATTAAATGTG 960
B_Florida_4_06_p20_egg_NA     881 TTGCCAGCAACAAAACCATAGAATGTGCTTGTAGAGATAACAGTTACACAGCAAAAAGACCCTTTGTCAAATTAAATGTG 960
                                  TTGCCAGCAACAAAACCATAGAATGTGCTTGTAGAGATAACAGTTACACAGCAAAAAGACCCTTTGTCAAATTAAATGTG

B_Florida_4_06_parent_NA      961 GAGACTGATACAGCGGAAATAAGATTGATGTGCACAGAGACTTATTTGGACACCCCCAGACCAAATGATGGAAGCATAAC 1040
B_Florida_4_06 p10 cell NA    961 GAGACTGATACAGCGGAAATAAGATTGATGTGCACAGAGACTTATTTGGACACCCCCAGACCAAATGATGGAAGCATAAC 1040
B_Florida_4_06_p10_egg_NA.seq 961 GAGACTGATACAGCGGAAATAAGATTGATGTGCACAGAGACTTATTTGGACACCCCCAGACCAAATGATGGAAGCATAAC 1040
B_Florida_4_06 p20 cell NA    961 GAGACTGATACAGCGGAAATAAGATTGATGTGCACAGAGACTTATTTGGACACCCCCAGACCAAATGATGGAAGCATAAC 1040
B_Florida_4_06_p20_egg_NA     961 GAGACTGATACAGCGGAAATAAGATTGATGTGCACAGAGACTTATTTGGACACCCCCAGACCAAATGATGGAAGCATAAC 1040
                                  GAGACTGATACAGCGGAAATAAGATTGATGTGCACAGAGACTTATTTGGACACCCCCAGACCAAATGATGGAAGCATAAC

B_Florida_4_06_parent_NA      1041 AGGGCCTTGCGAATCTGATGGGGACAAAGGGAGTGGAGGCATCAAGGGAGGATTTGTTCATCAAAGAATGGCATCCAAGA 1120
B_Florida_4_06 p10 cell NA    1041 AGGGCCTTGCGAATCTGATGGGGACAAAGGGAGTGGAGGCATCAAGGGAGGATTTGTTCATCAAAGAATGGCATCCAAGA 1120
B_Florida_4_06_p10_egg_NA.seq 1041 AGGGCCTTGCGAATCTGATGGGGACAAAGGGAGTGGAGGCATCAAGGGAGGATTTGTTCATCAAAGAATGGCATCCAAGA 1120
B_Florida_4_06 p20 cell NA    1041 AGGGCCTTGCGAATCTGATGGGGACAAAGGGAGTGGAGGCATCAAGGGAGGATTTGTTCATCAAAGAATGGCATCCAAGA 1120
B_Florida_4_06_p20_egg_NA     1041 AGGGCCTTGCGAATCTGATGGGGACAAAGGGAGTGGAGGCATCAAGGGAGGATTTGTTCATCAAAGAATGGCATCCAAGA 1120
                                   AGGGCCTTGCGAATCTGATGGGGACAAAGGGAGTGGAGGCATCAAGGGAGGATTTGTTCATCAAAGAATGGCATCCAAGA

B_Florida_4_06_parent_NA      1121 TTGGAAGGTGGTACTCTCGAACGATGTCTAAAACTAAAAGAATGGGGATGGGACTGTATGTAAAGTATGATGGAGACCCA 1200
B_Florida_4_06 p10 cell NA    1121 TTGGAAGGTGGTACTCTCGAACGATGTCTAAAACTAAAAGAATGGGGATGGGACTGTATGTAAAGTATGATGGAGACCCA 1200
B_Florida_4_06_p10_egg_NA.seq 1121 TTGGAAGGTGGTACTCTCGAACGATGTCTAAAACTAAAAGAATGGGGATGGGACTGTATGTAAAGTATGATGGAGACCCA 1200
B_Florida_4_06 p20 cell NA    1121 TTGGAAGGTGGTACTCTCGAACGATGTCTAAAACTAAAAGAATGGGGATGGGACTGTATGTAAAGTATGATGGAGACCCA 1200
B_Florida_4_06_p20_egg_NA     1121 TTGGAAGGTGGTACTCTCGAACGATGTCTAAAACTAAAAGAATGGGGATGGGACTGTATGTAAAGTATGATGGAGACCCA 1200
                                   TTGGAAGGTGGTACTCTCGAACGATGTCTAAAACTAAAAGAATGGGGATGGGACTGTATGTAAAGTATGATGGAGACCCA

B_Florida_4_06_parent_NA      1201 TGGACTGACAGTGAAGCCCTTGCTCTTAGTGGAGTAATGGTTTCGATGGAAGAACCTGGTTGGTATTCCTTTGGCTTCGA 1280
B_Florida_4_06 p10 cell NA    1201 TGGACTGACAGTGAAGCCCTTGCTCTTAGTGGAGTAATGGTTTCGATGGAAGAACCTGGTTGGTATTCCTTTGGCTTCGA 1280
B_Florida_4_06_p10_egg_NA.seq 1201 TGGACTGACAGTGAAGCCCTTGCTCTTAGTGGAGTAATGGTTTCGATGGAAGAACCTGGTTGGTATTCCTTTGGCTTCGA 1280
B_Florida_4_06 p20 cell NA    1201 TGGACTGACAGTGAAGCCCTTGCTCTTAGTGGAGTAATGGTTTCGATGGAAGAACCTGGTTGGTATTCCTTTGGCTTCGA 1280
B_Florida_4_06_p20_egg_NA     1201 TGGACTGACAGTGAAGCCCTTGCTCTTAGTGGAGTAATGGTTTCGATGGAAGAACCTGGTTGGTATTCCTTTGGCTTCGA 1280
                                   TGGACTGACAGTGAAGCCCTTGCTCTTAGTGGAGTAATGGTTTCGATGGAAGAACCTGGTTGGTATTCCTTTGGCTTCGA

B_Florida_4_06_parent_NA      1281 AATAAAAGATAAGAAATGTGATGTCCCCTGTATTGGGATAGAAATGGTACATGATGGTGGGAAAACGACTTGGCACTCAG 1360
B_Florida_4_06 p10 cell NA    1281 AATAAAAGATAAGAAATGTGATGTCCCCTGTATTGGGATAGAAATGGTACATGATGGTGGGAAAACGACTTGGCACTCAG 1360
B_Florida_4_06_p10_egg_NA.seq 1281 AATAAAAGATAAGAAATGTGATGTCCCCTGTATTGGGATAGAAATGGTACATGATGGTGGGAAAACGACTTGGCACTCAG 1360
B_Florida_4_06 p20 cell NA    1281 AATAAAAGATAAGAAATGTGATGTCCCCTGTATTGGGATAGAAATGGTACATGATGGTGGGAAAACGACTTGGCACTCAG 1360
B_Florida_4_06_p20_egg_NA     1281 AATAAAAGATAAGAAATGTGATGTCCCCTGTATTGGGATAGAAATGGTACATGATGGTGGGAAAACGACTTGGCACTCAG 1360
                                   AATAAAAGATAAGAAATGTGATGTCCCCTGTATTGGGATAGAAATGGTACATGATGGTGGGAAAACGACTTGGCACTCAG

B_Florida_4_06_parent_NA      1361 CAGCAACAGCCATTTACTGTTTAATGGGCTCAGGACAACTGCTGTGGGACACTGTCACAGGTGTTGATATGGCTCTGTAA 1440
B_Florida_4_06 p10 cell NA    1361 CAGCAACAGCCATTTACTGTTTAATGGGCTCAGGACAACTGCTGTGGGACACTGTCACAGGTGTTGATATGGCTCTGTAA 1440
B_Florida_4_06_p10_egg_NA.seq 1361 CAGCAACAGCCATTTACTGTTTAATGGGCTCAGGACAACTGCTGTGGGACACTGTCACAGGTGTTGATATGGCTCTGTAA 1440
B_Florida_4_06 p20 cell NA    1361 CAGCAACAGCCATTTACTGTTTAATGGGCTCAGGACAACTGCTGTGGGACACTGTCACAGGTGTTGATATGGCTCTGTAA 1440
B_Florida_4_06_p20_egg_NA     1361 CAGCAACAGCCATTTACTGTTTAATGGGCTCAGGACAACTGCTGTGGGACACTGTCACAGGTGTTGATATGGCTCTGTAA 1440
                                   CAGCAACAGCCATTTACTGTTTAATGGGCTCAGGACAACTGCTGTGGGACACTGTCACAGGTGTTGATATGGCTCTGTAA

B_Florida_4_06_parent_NA      1441 TGGAGGAATGGTTGAGTCTGTTCTAAACCCTTTGTTCCTATTTTGTTTGAACAATTGTCCTTACTGAGCTTAATTGTTTC 1520
B_Florida_4_06 p10 cell NA    1441 TGGAGGAATGGTTGAGTCTGTTCTAAACCCTTTGTTCCTATTTTGTTTGAACAATTGTCCTTACTGAGCTTAATTGTTTC 1520
B_Florida_4_06_p10_egg_NA.seq 1441 TGGAGGAATGGTTGAGTCTGTTCTAAACCCTTTGTTCCTATTTTGTTTGAACAATTGTCCTTACTGAGCTTAATTGTTTC 1520
B_Florida_4_06 p20 cell NA    1441 TGGAGGAATGGTTGAGTCTGTTCTAAACCCTTTGTTCCTATTTTGTTTGAACAATTGTCCTTACTGAGCTTAATTGTTTC 1520
B_Florida_4_06_p20_egg_NA     1441 TGGAGGAATGGTTGAGTCTGTTCTAAACCCTTTGTTCCTATTTTGTTTGAACAATTGTCCTTACTGAGCTTAATTGTTTC 1520
                                   TGGAGGAATGGTTGAGTCTGTTCTAAACCCTTTGTTCCTATTTTGTTTGAACAATTGTCCTTACTGAGCTTAATTGTTTC

B_Florida_4_06_parent_NA      1521 TGAAAAT 1528
B_Florida_4_06 p10 cell NA    1521 TGAAAAT 1528
B_Florida_4_06_p10_egg_NA.seq 1521 TGAAAAT 1528
B_Florida_4_06 p20 cell NA    1521 TGAAAAT 1528
B_Florida_4_06_p20_egg_NA     1521 TGAAAAT 1528
                                   TGAAAAT
```
